# How Efficient Are Isolation Protocols? Outcome of Isolation Protocol in Surgery during COVID-19 Pandemic: A Single Institute Experience

**DOI:** 10.1155/2023/5774071

**Published:** 2023-03-31

**Authors:** Rohan Chandra R. Gatty, Alphonsa Mary Job, Dinesh Shet

**Affiliations:** ^1^Department of Surgical Oncology, Father Muller Medical College Hospital, Mangalore 575 002, India; ^2^Department of General Surgery, Father Muller Medical College Hospital, Mangalore 575 002, India; ^3^Department of Medical Oncology, Father Muller Medical College Hospital, Mangalore 575 002, India

## Abstract

**Background:**

The timing of screening for SARS-CoV-2 preoperatively by RT-PCR/CBNAAT, isolation protocols in preoperative wards, operation theatres, and postoperative wards are not well established.

**Methods:**

Evaluating the effectiveness of maintaining three pathways of two COVID-19 negative pathways (1) immediate testing pathway (2) isolation, or quarantine for five days and testing prior to surgery pathway, and (3) the tested COVID-19-positive pathway, was the aim of the study. The primary objective was to assess the utility and outcome of the two COVID-19 negative pathways adopted before surgery in terms of infectivity (seroconversion; COVID-19 positivity rate before surgery and symptomatic COVID-19 disease after surgery). The secondary objective was to derive a practical protocol for isolation or quarantine for emergency and elective surgery. Enrolled patients were grouped based on the need for surgery; Group-1 emergency basis, Group-2 urgent basis, and Group-3 COVID-19 positive and the three channels were kept separate with separate dedicated healthcare staff for each channel.

**Results:**

There were 199 (4.56%) COVID-19-positive patients, of whom 80 (40%) were operated. COVID-19 positivity rate was low in Group 2 (3% vs. Group 1, 11%). There was no seroconversion from negative to positive in our patients during the peri-operative period.

**Conclusion:**

COVID-19 positivity rate in Group-2 was significantly less. None of the COVID-19-negative patients turned symptomatic and the probability of seroconversion from COVID-19-negative was less during the peri-operative period. The isolation protocol of non-COVID-19 positive patients with the separate channel is effective.

## 1. Introduction

The World Health Organization (WHO) declared coronavirus disease (COVID-19), an infectious disease caused by the severe acute respiratory syndrome coronavirus 2 (SARS-CoV-2) outbreak, a public health emergency of international concern on 30 January, 2020, and a pandemic on 11 March, 2020. As of 9 March, 2022, the pandemic had caused more than 450 million cases and 6.01 million deaths, making it the fifth deadliest in history [1]. India implemented a nation-wide lockdown from 22 March, 2020, and adopted various suppression and mitigation strategies through five-phased lockdowns [2], invoking a huge strain on the healthcare system, more so in the surgical field.

The effect of lockdown on the supply chain impacted the availability of healthcare accessories such as intensive care unit (ICU), beds, and ventilators. Elective and semi-emergency surgeries were stopped and opted for selectively in those with life-threatening complications/severe disease. As a result, there has been a dramatic shift in the professional framework of the healthcare system, and responsibilities. Screening for COVID-19, isolation, and quarantine were strictly followed to minimize contact and spread. As the associations globally have advised elective surgeries for COVID-19 infected, which extends from 4 to 12 weeks, patients have to bear long waiting period for surgeries, which is not a welcome news for oncology patients.

However, the timing of screening for SARS-CoV-2 in preoperative surgical patients by real time polymerase chain reaction (RT-PCR)/cartridge-based nucleic acid amplification test (CBNAAT), and isolation protocols in preoperative surgical wards, operation theatres, and postoperative wards are not well defined and data is insufficient to support their effectiveness [3–6]. Hence, there is a need to have evidence to arrive at concrete conclusions.

This study was undertaken to assess the outcome of using separate channels for COVID-19-positive and -negative patients, the impact of isolation on COVID-19 status in the perioperative period among the patients who required emergency surgical procedures.

## 2. Materials and Methods

This was a time bound observational prospective study performed during the first wave of the COVID-19 pandemic from June 01, 2020, to November 30, 2020, after obtaining approval from the Institutional Ethics Committee for Biomedical Health Research. Evaluating the effectiveness of maintaining three pathways of two COVID-19-negative pathways, namely, (1) immediate testing pathway (2) isolation, or quarantine for five days and testing prior to surgery pathway, and (3) tested COVID-19-positive pathway during the pandemic was the aim. The objectives of the study were to assess the utility and outcome of two COVID-19-negative pathways adopted before surgery in terms of infectivity (seroconversion; COVID-19 positivity rate before surgery and symptomatic COVID-19 disease after surgery) and impact of the three pathways adopted for COVID-19 during postsurgery in deriving a practical protocol for isolation or quarantine for emergency and elective surgery during the pandemic period.

Patients who had to undergo surgery and willing to undergo RT-PCR or CBNAAT for the detection of COVID-19 were included in the study. All enrolled patients underwent surgery following the COVID-19 isolation protocol devised by the COVID-19 TASK FORCE in the hospital after collecting various inputs from the professional surgical associations.

Enrolled patients were grouped into the following groups:

  Group 1: patients who had to undergo surgery on an emergency basis within 24–48 hrs because of the seriousness of the surgical disease/condition. They were admitted to the COVID-19 screening ward, or in a separate room.
  Group 2: patients who had to undergo surgery on an urgent basis (cancer or a benign condition having a complication) were isolated for 5 days in the hospital (this was based on the concept that the virus had a 5 days incubation period to get detected in the RT-PCR test) either in a private room or on the fifth day of testing in preoperative ward.
  Group 3: all COVID-19-positive patients requiring surgery.

However, a few patients who needed surgery on an urgent basis but not on emergency basis preferred to be hospitalised in Group 1 immediate surgery groiup) and were allotted the same.

**Figure 1:**
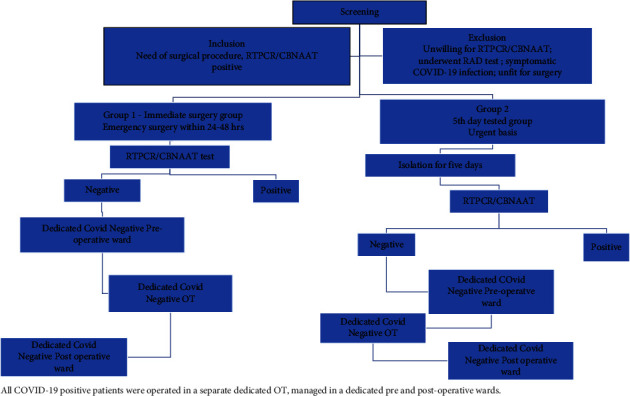
Three channel surgical protocol.

A schematic representation of the three channels is given in Figure 1. Patients who tested and turned positive for COVID-19 but remained asymptomatic were operated in the emergency group but not in the urgent group, and were operated in the dedicated COVID-19-positive operation theatre meant for this COVID-19-positive surgery group. After surgery, these patients were managed in the COVID-19-positive postoperative ward. All three channels were kept separate to avoid any mix of patients in different groups. Separate dedicated healthcare staff for each channel were allotted.

Our institution, a 1200 bedded tertiary care hospital, has six operation theatres (OT) with adjoining six postoperative blocks. Three OT blocks and the adjoining postoperative wards were converted into (a) immediate tested OT and postoperative ward, (b) 5^th^ day tested OT and postoperative ward, and (c) the COVID-19, positive OT, and postoperative ward.

The three groups had dedicated separate preoperative-wards, OTs, and postoperative wards in different areas of the hospital. Patients staying in a private room were managed preoperatively in their rooms and postoperatively in the respective dedicated postoperative wards and later in their private room.

## 3. Results

Of the 4365 hospitalised surgical inpatients during the study period, 199 (4.56%) tested positive for COVID-19. There were 101 (51%) females and 98 (49%) males. Patients aged 21–40 years comprised of 52% of the COVID-19-positive study population. Age-wise and gender-wise distribution of the study population is provided in Tables 1 and 2.

Of the 199 COVID-19-positive patients, 80 underwent various surgical procedures.  Table 3 details the COVID-19-positive cases, inclusive of cancer patients reported during the study period from various departments. Patients from the department of general surgery (42.5%) comprised the highest proportion followed by obstetrics and gynecology (27.5%).

overall COVID-19 positivity rate was low among the 5-day group (3%) compared to the immediate surgery group (11.2%) though the rate was high in the first month during the study (5-day vs. immediate surgery group, 1.18% vs. 0.67%), and no significant difference in the last month (5-day vs. immediate surgery group 4.76% vs. 4.48%) (Table 4). There was no drastic change in the COVID-19 positivity rate in the 5-day group, while there was a fluctuation in the immediate surgery group.

**Figure 2:**
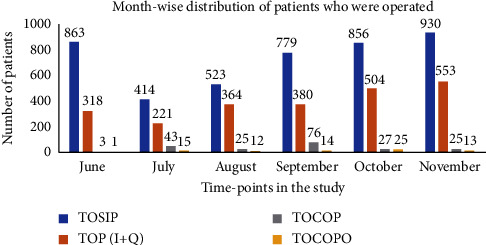
Comparison of various categories of patients operated during the study period. TOSIP = total surgical inpatients; TOP (+Q) = total operated patients; TOCOP = total COVID-19 positive patients; TOCOPO = total operated COVID-19 positive patients.

Figure 2 compares various categories of patients who required surgery.

COVID-19 positivity rate was high during July, 2020, (10.39%) and September, 2020 (9.75%). The COVID-19 positivity rate among the study population reflected the disease burden, high during the initial study period, and showed a gradual declining trend in the later period (Table 5).  Table 6 compares the total and operated COVID-19 patients during the study period; the proportion of COVID-19 positive patients who underwent surgery was high during October, 2020, (92.60%) and November, 2020 (52%). The proportion of COVID-19 patients who were operated increased gradually from one of three patients to 25/27 in October 2020 and 13/25 in November 2020.

Table 7 compares various parameters between the two study groups; there was a statistically significant difference (*p* < 0.001) in the CBNAAT test and the number of patients operated. CBNAAT testing was significant in the immediate surgery group while, RTPVR was significant in the 5-day group.

## 4. Discussion

COVID-19 pandemic taught us to prioritise the events including surgery; lifesaving procedures were considered despite the risk to healthcare professionals, but elective surgeries took a backseat, affecting a large number of needy patients. The guidelines delineate the timelines for elective surgeries and for COVID-19-positive patients [7]. Delaying elective surgeries is the protocol followed globally [8, 9]. Current guidelines advise delaying elective surgeries, as high rate of mortality is reported in the first month of surgery among those with COVID-19 infection than those without [10]; an international multicentric study has observed that delaying elective surgeries for ≥7 weeks is associated with a better outcome [11]. Though a safe option, postponing elective surgery does not hold good for emergency surgery, particularly for cancer patients, in whom delaying may have an immense tumor effect on the patients, increasing the mortality (4–8%) [12]. Hence, delaying surgery is not a viable option and is not justifiable in a few life-threatening conditions.

A clinical guide for the management recommends a thorough risk assessment and COVID-19-free sites for negative patients. COVID-19 testing was to be done 48 hours before surgery, self-isolated for seven days before hospitalization. If the patient turns COVID-19-positive during hospitalization, they are to be operated in the COVID-19-positive facility [13]. Testing patient before surgery is a crucial step. RTPCR/CBNAAT tests are the approved, standard tests for detecting COVID-19 infection, which is done before considering the patient for surgery. Hence, we attempted to detect the necessity to isolate the patients, made them to wait till the completion of the average incubation period of SARS-CoV-2, and tested using RT-PCR, if asymptomatic for COVID-19 on admission.

### 4.1. Testing before Surgery for COVID-19

A systemic meta-analysis has proved RT-PCR as the gold standard test for COVID-19 [14, 15] to acquire reliable results [16, 17], and is included in the routine panel of investigations [18, 19]. Most of the academic centres are using RT-PCR for screening [20] and is recommended before surgeries [21]. The ideal time for screening is 48–72 hours before surgery. Antigen testing and other tests are not preferred for surgical patients as the sensitivity is lower than that of RT-PCR [15]. Hence, we used RT-PCR and CB NAAT for screening these patients.

### 4.2. Need for Separate Zones for COVID-19 Patients

Several protocols and techniques have been detailed but each with practical difficulties. Separating COVID-19 and non-COVID-19 zones have been practiced [22]; Coleman et al. [23] categorized the patients into baseline, intermediate, and high-risk groups and managed these patients in dedicated surgical intensive care units to minimize the spread of COVID-19 infection within the hospital. For those requiring surgery, a 3-point screening method was followed preoperatively to ensure a COVID-19-free environment: telephonic screening for symptoms for 3 days and one day before surgery, and on the day of surgery. Despite prior testing, many healthcare workers contacted COVID-19 infection, further raising the concern of spread. Hence, we channelised our patients into three separate streams from preoperative to postoperative period, to prevent patients of different categories coming in contact, and to curtail the spread of infection.

### 4.3. Roadmap towards Separate Zones

Brindle et al. [24] suggest maintaining a dedicated OT with a postoperative care facility for COVID-19-positive patients, from where the patient to be shifted to a dedicated ICU or their respective ward. Similar suggestions were given by Coccolini et al. [25] We followed the same, which helped and proved beneficial in minimizing the spread of infection.

Separating the OT complex into separate zones (entry, changing rooms at entry and exit, OT, and exit room) [26] has been recommended and a similar roadmap was given by Awad et al. [27] As spread of infection through anesthetic equipment is a concern, use of appropriate precautionary measures (ex. filters) is necessary during each use [28, 29]. We had separate OT complexes with appropriate precautionary measures for COVID-19-positive and negative patients, without any overlapping.

We used a unique 3-tier system for three different groups, which helped us to continue the surgeries successfully, particularly the emergency surgeries for cancer patients. Ours is the first study from India to use dedicated channels, OTs, and staffs for three different groups. An extensive literature survey did not yield similar studies for comparison of our results. Few studies have followed the prescreening tests, but none have experimented with 5 day observation period, and repeat COVID-19 testing preoperatively. There is no study available at the time this manuscript was written that has documented the impact of the waiting period for RT-PCR before surgery, immediate testing and delayed testing with RTPCR in the asymptomatic surgical patients and their chances of becoming symptomatic for COVID-19 in the postoperative period. Our study supports immediate and delayed testing with RTPCR/CBNAAT in the pre-operative period for serological surveillance, and the probability of seroconversion to COVID-19-positive during the peri-operative period is less.

### 4.4. Basis for Prioritization of Patients

We categorized the patients on a priority basis; those who needed surgery within the first 24 hours were given the highest priority and were catered to. It is well established that COVID-19 is more contagious during the symptomatic phase, but the contribution of asymptomatic patients is not less in the spread and cannot be ignored. Hence, we gave a five-day observation (to allow the patients with the viral load who were asymptomatic on admission with an assumption that they may later turn symptomatic or become asymptomatic positive during the incubation period of 5 days) to those who had an urgent need, but could bear the waiting period of five days, which was used to note symptom development. In our study, none of the asymptomatic COVID-19 patients who were operated in the defined isolation pathways became symptomatic in the postoperative period, reinstating that the defined isolation pathway is useful in creating a safe surgical environment against COVID-19, both for the patient and healthcare workers.

There is clinically no window period for the asymptomatic COVID-19-positive patients to get detected on RT-PCR; otherwise, a few patients in this group would have turned positive in the postoperative period. In our study, the positivity rate was high in the immediate group (11% vs. 3%).

### 4.5. Seropositivity for COVID-19 and Surgery

Of COVID-19-positive surgical patients (4.56%) of whom 40.20% were operated, which was done uninterruptedly by following separate routes, surgery was performed in >30% of the COVID-19-positive surgical inpatients throughout the study period, except during September 2020. We propose that it is not necessary to wait for 5 days (incubation period) prior to surgery for the RT-PCR test. Screening asymptomatic COVID-19 patients planned for surgery can be performed safely in the immediate testing isolation pathway with RT-PCR. The positivity rate was consistently low in the 5-day group even during the peak of the COVID-19 wave. However, more evidence is needed in this prospect.

### 4.6. Importance of Isolating/Quarantining the Surgical Patients

The concept of group 2, i.e., testing after 5 days of quarantine, was based on the concept of the incubation/window period of the virus. The incubation period is the basis for quarantine in infections to disrupt the spread; the median incubation time of SARS- COV- 2 is 5.1 days; available data records that symptoms of COVID-19 are seen around 11.5 days in 97.5% and may extend up to 14 days [30, 31]. A meta-analysis has shown that the incubation period can be between 6 and 7 days with geographic variation [32].

In our study, we observed that the positivity rate before surgery was high in the immediate tested isolation pathway when compared with the 5^th^ day tested isolation pathway (11% vs. 3%). The positivity rate was consistently low in the 5 days group even during the peak of the COVID- 19 wave. The probable reason would be that these patients got isolated from the wave, when the wave was prevalently flowing in the community. Our results indicate that this 5 -days quarantine pathway (based on the incubation period of the virus) definitely helps during the peak period of community transmission in a pandemic outbreak. However, more concrete studies are required to support our observation.

In our study, patients who underwent surgery after testing negative in either of the pathways (immediate or 5 day quarantine) never became symptomatic for COVID-19 in the postoperative period. Hence, our observation suggests that either of the isolation pathway protocols is equally effective in screening for COVID-19 before surgery and patients can be safely operated without concern of them becoming symptomatic for COVID-19 in the postoperative period.

We recommend that asymptomatic patients planned for surgery during a pandemic outbreak can be operated safely in either of the pathways, the need to get quarantined for 5 days before getting tested for COVID-19 may not be required routinely; during the peak period of the outbreak (community transmission phase), the 5 day quarantine and testing would be helpful in getting more patients to undergo essential surgery, as the chances of seroconversion to COVID-19 positive will be negligible. However, further studies are needed to understand this concept, and for stronger evidence.

Gaps exist in various protocols experimented in surgery during the COVID-19 pandemic that needs to be bridged. We observed that our protocol is practical, ideal, and reduces the hospital cross-infection. With the appearance of new waves, it is imperative that the existing hospital protocols be updated to handle the health emergency. Isolation surgical protocols have to be diligently implemented to minimize the spread of infection and to be followed meticulously. Observations made from this pandemic should be accounted as it will be useful in handling healthcare crisis during pandemics in future, if any occurs.

## 5. Conclusion

Preoperative COVID-19, testing is advised as it helps to categorise the patients on seropositivity. Immediate and delayed testing with RTPCR/CBNAAT in the preoperative period is helpful in detecting the serological status/surveillance. COVID-19 positivity rate in the 5-days quarantined group was significantly less than those in the immediate tested group. There is no need to quarantine asymptomatic patients in the hospital for three to five days before testing, as none of the patients who were COVID-19 negative in the preoperative period turned symptomatic in the postoperative period either in the immediate tested or 5 day quarantined group. Our results suggest that the probability of seroconversion from COVID-19 negative to positive is less during the peri-operative period.

The isolation protocol of all non-COVID-19 positive patients with separate channel is effective, and none of the operated COVID-19-negative patients became positive during their hospital stay.

## Figures and Tables

**Table 1:** Age-wise distribution of the study population who were COVID-19 positive.

Month	*Age group (years)*							Total
	21–30	31–40	41–50	51–60	61–70	71–80	81–90	
June	02 (1%)	10 (0.5%)	—	—	—	—	—	03 (1.5%)
July	15 (7.5%)	08 (4%)	04 (2%)	09 (4.5%)	05 (2.5%)	01 (0.5%)	01 (0.5%)	43 (21.6%)
August	10 (5%)	04 (2%)	06 (3%)	03 (1.5%)	02 (1%)	—	—	25 (12.5%)
September	21 (10.6%)	12 (6%)	15 (7.5%)	08 (4%)	13 (6.5%)	07 (3.5%)	—	76 (38%)
October	10 (5%)	06 (3%)	03 (1.5%)	04 (2%)	02 (1%)	01 (0.5%)	01 (0.5%)	27 (13.6%)
November	06 (3%)	09 (4.5%)	02 (1%)	04 (2%)	03 (1.5%)	01 (0.5%)	—	25 (12.5%)
Total	64 (32%)	40 (20%)	30 (15%)	28 (14%)	25 (12.5%)	10 (5%)	02 (1%)	199 (100%)

**Table 2:** Gender-wise distribution of the study population who were COVID-19 positive.

Month	Male	Female	Total
June	0	03 (1.5%)	03 (1.5%)
July	12 (6%)	31 (15.5%)	43 (21.6%)
August	12 (6%)	13 (6.5%)	25 (12.5%)
September	47 (23.6%)	29 (14.6%)	76 (38%)
October	10 (5%)	17 (8.5%)	27 (13.6%)
November	17 (8.5%)	08 (4%)	25 (12.5%)
Total	98 (49%)	101 (51%)	199 (100%)

**Table 3:** Department-wise COVID-19 positive cases.

Department	Category	June	July	August	September	October	November	Total
Obstetrics and gynecology	Covid +ve	1	10	2	2	5	2	22
	Cancer cases	3	1	2	2	0	2	10
	Total cases	77	62	50	67	95	107	458

Surgical gastroenterology	Covid +ve	0	0	3	3	2	0	08
	Cancer cases	7	2	3	6	11	5	34
	Total cases	13	5	6	17	25	16	82

General surgery	Covid +ve	0	3	5	5	13	8	34
	Cancer cases	3	4	2	11	17	19	56
	Total cases	49	35	43	66	110	119	422

Otolaryngology	Covid +ve	0	0	0	0	0	0	0
	Cancer cases	1	0	0	0	5	3	09
	Total cases	6	2	8	8	16	26	66

Surgical oncology	Covid +ve	0	1	1	3	0	0	05
	Cancer cases	38	12	20	43	60	50	223
	Total cases	38	12	20	43	60	50	223

Other departments^*∗*^	Covid +ve	0	1	1	1	5	3	11

Total cases		183	116	127	201	306	318	1251
Total COVID-19 +ve operated		01	15	12	14	25	13	80
Total cancer cases		52	19	27	62	93	79	332

^∗^Plastic surgery, ophthalmology, urology, orthopedics, cardiothoracic vascular surgery, neuro surgery, and paediatric surgery.

**Table 4:** COVID-19 positive patients in 5-day and immediate groups preoperatively.

Month/2020	*Immediate group*			*5-day group*		
	COVID-19 +ve	Total	Positivity %	COVID-19 +ve	Total	Positivity %
June	1	149	0.67	2	169	1.18
July	41	114	35.96	2	107	1.87
August	17	175	9.71	8	189	4.23
September	70	228	30.70	6	152	3.95
October	25	416	6.00	2	88	2.27
November	22	490	4.48	3	63	4.76
Total	176	1572	11.2	23	768	3

**Table 5:** COVID-19 positivity rate among the surgical patients.

Month/2020	TOSIP	TOCOP	Surgical IP COVID-19 positivity rate (%)
June	863	3	0.35
July	414	43	10.39
August	523	25	4.78
September	779	76	9.75
October	856	27	3.15
November	930	25	2.69
Total	4365	199	4.5

TOSIP = total surgical inpatients; TOCOP = total operated COVID-19 positive patents.

**Table 6:** Operative percentage of COVID-19 positive patients.

Month/2020	TOCOP	TOCOPO	Operative (%) COVID-19 positive patients	Surgical IP COVID-19 positive rate (%)
June	3	1	33.33	0.35
July	43	15	34.88	10.39
August	25	12	48	4.78
September	76	14	18.42	9.75
October	27	25	92.60	3.15
November	25	13	52.00	2.69
Total	199	80		

TOCOP = total COVID-19 positive patients; TOCOPO = total number of COVID-19 patients operated.

**Table 7:** Comparison of various parameters between the two study groups.

Parameters	*Group 1 (n* *=* *1572) (0 day)*		*Group 2 (n* *=* *768) 5 day*		*P* value
	Positive	Negative	Positive	Negative	
CBNAAT	176	1,396	0	0	0.000^*∗*^
RT-PCR	0	0	23	745	
Operated	80	1,401	0	740	0.000^*∗*^
Not operated	91	0	28	0	
Oncology	0	100	05	223	
Non-oncology	0	0	0	0	

RT-PCR = real time polymerase chain reaction. CBNAAT = cartridge based nucleic acid amplification test. ^*∗*^ Statistically significant.

## Data Availability

The data used to support the findings of this study will be made available upon request to Dr Shet.
